# Pilot Implementation of a Remote Primary Care and Psychiatry Model (MAP-PSI) for Youth Depression in Rural and Indigenous Mexican Communities

**DOI:** 10.1192/j.eurpsy.2026.11356

**Published:** 2026-07-31

**Authors:** L. Díaz-Castro, G. B. Ramírez-Rodríguez

**Affiliations:** 1 Department of Epidemiological and Psychosocial Research; 2 Laboratory of Neurogenesis, Division of Clinical Research, National Institute of Psychiatry Ramón de la Fuente Muñiz, Mexico City, Mexico

## Abstract

**Introduction:**

Youth depression represents a growing burden in rural and Indigenous communities, where access to mental health services is scarce. There is an urgent need for scalable, culturally adapted, and community-based models to improve detection and care.

**Objectives:**

To evaluate the feasibility and effectiveness of the MAP-PSI (Primary Care and Psychiatry Model), a telemedicine-based intervention designed to reduce depressive symptoms among youth aged 15–25 in marginalized settings.

**Methods:**

A quasi-experimental pre-post study was conducted (March 2023 – March 2025) in Ciudad Fernández, San Luis Potosí, Mexico. The intervention combined: (1) bilingual psychoeducation, (2) brief cognitive-behavioral therapy (CBT), and (3) psychiatric care via telemedicine. Screening instruments included the PHQ-9 and KAP questionnaires. A total of 1,057 youth were screened, and 400 received stepped-care based on symptom severity. Data were analyzed using non-parametric tests (Wilcoxon, Mann-Whitney U).

**Results:**

Analysis of the results showed a 60% average reduction in depressive symptoms across modalities (p < .001). Also, 25 cases of suicidal ideation were successfully treated, and 34,224 school absence days were prevented (84% reduction). Moreover, the intervention reached over 1,500 youth with >85% adherence and high community acceptability. The pilot implementation achieved a 98% reduction in out-of-pocket expenses. This pilot study involved 67 bilingual community health promoters who were trained and certified.

**Image 1:**
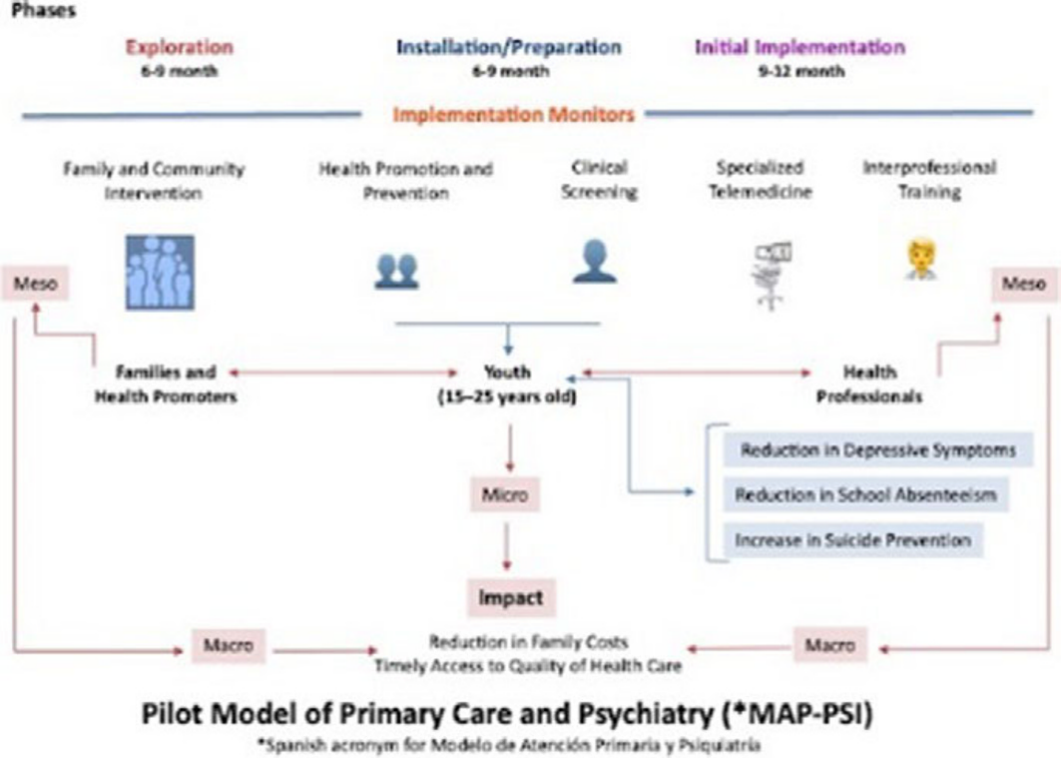
Image 1: Long description.

**Conclusions:**

The MAP-PSI model proved to be a culturally relevant, cost-effective, and feasible strategy for youth depression in underserved regions. Its stepped-care, telemedicine-supported approach has strong potential for replication in other low-resource settings. Further studies with controlled designs are warranted to evaluate long-term outcomes.

**Disclosure of Interest:**

None Declared

## Long descriptions

### Image 1: Long description

The flowchart is organized into three horizontal phases at the top.

1. Exploration (6 to 9 months) includes Family and Community Intervention.

2. Installation or Preparation (6 to 9 months) includes Health Promotion and Prevention and Clinical Screening.

3. Initial Implementation (9 to 12 months) includes Specialized Telemedicine and Interprofessional Training.

Below these phases, a horizontal line labeled Implementation Monitors connects to a central node labeled Youth (15 to 25 years old). This central node is linked via bidirectional arrows to Families and Health Promoters on the left and Health Professionals on the right. Both side groups are labeled as Meso level.

A blue arrow from the Youth node points to a list of outcomes: Reduction in Depressive Symptoms, Reduction in School Absenteeism, and Increase in Suicide Prevention.

Vertically below the Youth node, the flow moves to Micro level and then to Impact. The Impact section at the bottom is flanked by Macro level labels and lists two primary results: Reduction in Family Costs and Timely Access to Quality of Health Care. The entire diagram is titled Pilot Model of Primary Care and Psychiatry (*MAP-PSI).

Navigate back to Image 1:.

